# Effects of Ultramicronized Palmitoylethanolamide on Mitochondrial Bioenergetics, Cerebral Metabolism, and Glutamatergic Transmission: An Integrated Approach in a Triple Transgenic Mouse Model of Alzheimer's Disease

**DOI:** 10.3389/fnagi.2022.890855

**Published:** 2022-05-24

**Authors:** Francesco Bellanti, Vidyasagar Naik Bukke, Archana Moola, Rosanna Villani, Caterina Scuderi, Luca Steardo, Gianmauro Palombelli, Rossella Canese, Sarah Beggiato, Mario Altamura, Gianluigi Vendemiale, Gaetano Serviddio, Tommaso Cassano

**Affiliations:** ^1^Department of Medical and Surgical Sciences, University of Foggia, Foggia, Italy; ^2^Department of Physiology and Pharmacology “V. Erspamer”, Sapienza University of Rome, Rome, Italy; ^3^MRI Unit Core Facilities, Istituto Superiore di Sanità, Rome, Italy; ^4^Department of Life Sciences and Biotechnology, University of Ferrara, Ferrara, Italy; ^5^Department of Clinical and Experimental Medicine, University of Foggia, Foggia, Italy

**Keywords:** glutamate, mitochondria, hippocampus, frontal cortex, microdialysis, Alzheimer's disease

## Abstract

The therapeutic potential of ultramicronized palmitoylethanolamide (um-PEA) was investigated in young (6-month-old) and adult (12-month-old) 3 × Tg-AD mice, which received um-PEA for 3 months *via* a subcutaneous delivery system. Mitochondrial bioenergetics, ATP homeostasis, and magnetic resonance imaging/magnetic resonance spectroscopy were evaluated in the frontal cortex (FC) and hippocampus (HIPP) at the end of um-PEA treatment. Glutamate release was investigated by *in vivo* microdialysis in the ventral HIPP (vHIPP). We demonstrated that chronic um-PEA treatment ameliorates the decrease in the complex-I respiration rate and the FoF1-ATPase (complex V) activity, as well as ATP content depletion in the cortical mitochondria. Otherwise, the impairment in mitochondrial bioenergetics and the release of glutamate after depolarization was not ameliorated by um-PEA treatment in the HIPP of both young and adult 3 × Tg-AD mice. Moreover, progressive age- and pathology-related changes were observed in the cortical and hippocampal metabolism that closely mimic the alterations observed in the human AD brain; these metabolic alterations were not affected by chronic um-PEA treatment. These findings confirm that the HIPP is the most affected area by AD-like pathology and demonstrate that um-PEA counteracts mitochondrial dysfunctions and helps rescue brain energy metabolism in the FC, but not in the HIPP.

## Introduction

Alzheimer's disease (AD) is a consequence of several detrimental processes, such as protein aggregation, oxidative stress, mitochondrial malfunction, and neuroinflammation, finally resulting in the loss of neuronal functions (Blass and Gibson, 1991; Sullivan and Brown, 2005; Yao et al., 2009; Querfurth and Laferla, 2010; Serviddio et al., 2011; Cassano et al., 2016, 2019; Carapelle et al., 2020). Starting from this evidence, multitarget drugs have been increasingly sought after over the last decades (Barone et al., 2019; Cassano et al., 2019). In this regard, palmitoylethanolamide (PEA) seems to exert neuroprotective effects by modulating more therapeutic targets at the same time (Valenza et al., 2021). In fact, it has been demonstrated that PEA, the naturally occurring amide of ethanolamine and palmitic acid, is an endogenous lipid that exerts its beneficial effects through the peroxisome proliferator-activated receptors (PPARs) involvement, transient receptor potential vanilloid type 1 channel, orphan G-protein-coupled receptor 55, and the so-called entourage effect on the endocannabinoid system (Devchand et al., 1996; Delerive et al., 2001; LoVerme et al., 2005, 2006; Scuderi et al., 2011; Tomasini et al., 2015; Bronzuoli et al., 2019; Beggiato et al., 2020a,b). In addition to its known anti-inflammatory activity, PEA protects cultured mouse cerebellar granule cells from glutamate toxicity and reduces histamine-induced cell death in hippocampal cultures (Skaper et al., 1996).

Furthermore, it has been demonstrated that PEA exerts *in vitro* and *in vivo* a combination of neuroprotective and anti-inflammatory effects in amyloid-β (Aβ)-induced toxicity by interacting at the PPAR-α nuclear site (D'Agostino et al., 2012; Scuderi et al., 2012, 2014). The neuroprotective action of PEA has been established also in transgenic animal models of AD (D'Agostino et al., 2012; Bronzuoli et al., 2018; Scuderi et al., 2018). In fact, we have previously demonstrated that ultramicronized PEA (um-PEA), a formulation endowed with better bioavailability, can rescue learning and memory impairments in a triple transgenic mouse model of AD (3 × Tg-AD) by exerting anti-inflammatory properties, dampening reactive astrogliosis, and promoting the glial neurosupportive function. Moreover, um-PEA strongly suppresses Aβ(1–42) expression and reduces the abnormal phosphorylation of tau in 3 × Tg-AD mice (Scuderi et al., 2018).

Mitochondrial dysfunctions are related to inflammation and other energy-dependent disturbances, where the generation of reactive oxygen species (ROS) exceeds the physiological antioxidant activity, causing cellular oxidative damage (Lin and Beal, 2006; Reddy, 2009; Romano et al., 2017). Mitochondrial dysfunction can lead to glutamatergic neurotransmission alterations (Vos et al., 2010) and excitotoxicity (Schinder et al., 1996). In fact, excessive glutamate concentration can cause excitotoxicity, leading to calcium influx, mitochondrial dysfunction, and subsequent cell death (Mahmoud et al., 2019). Therefore, considering the tight link between mitochondria and glutamate, it could be crucial to investigate their alterations in the context of AD pathology.

In the last decade, neuroimaging has been used to complement clinical assessments in the early detection of AD, showing that morphological change (such as measurement of the hippocampal volume) might be an important hallmark for the diagnosis of AD. More recently, the development of magnetic resonance spectroscopy (MRS) in the field of AD revealed that tremendous metabolic changes occur during the progression of AD correlating with cognitive abnormalities (Meyerhoff et al., 1994; Shonk et al., 1995; Frederick et al., 1997; Rose et al., 1999; Kantarci et al., 2000; Huang et al., 2001). Nevertheless, few studies with relatively small sample sizes investigated MR spectral profile alteration as a biomarker for treatment response in AD, and some of them correlated this alteration with psychiatric symptoms in patients with AD (Sweet et al., 2002; Bartha et al., 2008). Although metabolite abnormalities in AD were demonstrated in different samples and pathologically confirmed cases, the pathological significance of these changes is not fully understood. Therefore, in our study, we exploited the availability of the MRS to investigate whether it can provide complementary predictive information regarding (i) progression of the AD-like pathology and (ii) um-PEA treatment effects.

In this article, we used the 3 × Tg-AD mice harboring three mutant human genes (betaAPPSwe, PS1M146V, and tauP301L) to directly test the hypothesis that (i) progressive accumulation of Aβ is closely related to mitochondrial, metabolic, and glutamatergic alterations, and (ii) chronic um-PEA administration may ameliorate such alterations. To test these hypotheses, we used an integrated approach, based on *ex vivo* studies of several mitochondrial bioenergetic parameters, *in vivo* studies of magnetic resonance imaging (MRI) and spectroscopy (MRS) to evaluate brain metabolites, as well as neurochemical analysis of extracellular levels of glutamate by microdialysis in 3 × Tg-AD vs. non-Tg control mice tested at two different stages (mild and severe) of AD-like pathology and subcutaneously treated for 3 months with um-PEA.

## Materials and Methods

### Animals and Treatment

A total of 3 × Tg-AD male mice and their sex- and age-matched wild-type littermates (non-Tg) (C57BL6/129SvJ) were maintained in controlled conditions (12 h light/12 h dark cycle, temperature 22°C, humidity 50–60%, fresh food, and water *ad libitum*). All procedures were conducted in accordance with the guidelines of the Italian Ministry of Health (D.L. 26/2014) and the European Parliamentary directive 2010/63/EU. All efforts were made to minimize the number of animals used and their suffering. In addition, 3- and 9-month-old mice were subcutaneously implanted with a 90-day-release pellet containing either 28 mg of um-PEA (Innovative Research of America, Sarasota, Florida; cat# NX-999) or vehicle (cat# NC-111), as previously described (Scuderi et al., 2018). Briefly, an um-PEA or a vehicle pellet was surgically positioned in a subcutaneous pocket created with a blunt probe between the shoulder blades. um-PEA (EPT2110/1) was obtained from the Epitech group (Saccolongo, Italy), and both dosage and administration routes were chosen according to previous data (Costa et al., 2002; Grillo et al., 2013; Scuderi et al., 2018). Both non-Tg and 3 × Tg-AD mice were randomly assigned to either vehicle or um-PEA group. At the end of the 90-day treatment, mitochondrial bioenergetics, MRI/MRS experiments, and microdialysis/high-performance liquid chromatography (HPLC) analysis were performed as previously described (Cassano et al., 2012; Scuderi et al., 2018).

### Mitochondrial Bioenergetics and ATP Homeostasis

On the day of the experiment, 6- and 12-month-old mice were decapitated and brains were rapidly removed and placed on a cold surface to dissect the hippocampus (HIPP), frontal cortex (FC), and cerebellum (Cassano et al., 2012). Experiments were conducted on four samples per group per each brain area. To obtain an optimal sample amount, tissues from three animals were pooled (a total of 12 mice per group). Mitochondria were freshly isolated by using a gradient of Percoll, as previously described (Cassano et al., 2012). Briefly, brain areas were weighted (100 mg) and immersed in ice-cold mitochondrial isolation buffer (MIB) containing 0.25 M sucrose, 0.5 mM K-EDTA, and 10 mM Tris–HCl (pH 7.4). Each sample was homogenized in 3.8 ml of 12% Percoll in MIB (5% w/v) using Dounce homogenizers with glass pestles. In addition, 3 ml of homogenate was then layered onto a previously poured, 3.5 ml 26% Percoll, on 3.5 ml 40% Percoll density gradient. The gradient was centrifuged at 19,000 rpm (30,000 × *g*) in a Sorval RC-5B type rotor for 5 min. The resulting top layer containing myelin and other cellular debris was carefully removed using a Pasteur pipette and discarded. Fraction 2 containing the mitochondria was removed by pipetting with a 200 ml gel loading tip and diluted 1:4 in cold MIB and centrifuged at 14,000 (15,000 × *g*) rpm for 10 min. The resulting pellet was then resuspended in 1 ml of MIB and centrifuged at 14,000 rpm (15,000 × *g*) for 5 min. The final pellet was resuspended in 100 ml of MIB containing 10% 10 mg/ml bovine serum albumin. After isolation, mitochondria were assayed for oxygen consumption at 37°C in a thermostatically controlled oxygraph apparatus equipped with Clark's electrode and a rapid mixing device (Hansatech Instruments, Ltd., Norfolk, England, UK). Mitochondrial respiration was triggered by glutamate and malate as complex I-linked substrates, or succinate (in the presence of rotenone as complex I inhibitor) as complex II-linked substrate. Oxygen uptake in states 3 and 4 and respiratory control index (RCI) was calculated as previously reported (Cassano et al., 2012). Respiratory activity was calculated as oxygen nmol per minute per mg of protein.

The F_O_F_1_-ATPase activity was measured following ATP hydrolysis with an ATP-regenerating system coupled to nicotinamide adenine dinucleotide phosphate (NADPH) oxidation, as previously reported (Cassano et al., 2012).

Measurement of ATP concentration in the total homogenates and mitochondrial fractions from brain areas was performed by using a commercial bioluminescent assay kit (Sigma-Aldrich, St. Louis, MO, USA).

### Magnetic Resonance Imaging and Spectroscopy

The 6- and 12-month-old mice (*n* = 5–7) underwent MRI/MRS scanning to evaluate genotype- and treatment-induced differences in brain metabolites of FC and HIPP. MRI/MRS analyses were conducted at 4.7 T on a Varian/Agilent Inova horizontal bore system (Agilent, Palo Alto, USA) using a combination of volume and surface coil (RAPID Biomedical, Rimpar, Germany) according to a protocol described in Scuderi et al. (2018). Briefly, mice were anesthetized with isoflurane (IsoFlo, Abbott SpA, Berkshire, UK) 1.5–2.5% in O_2_ 1 L/min. Anatomical T2-weighted sagittal MRIs were acquired for the positioning of the voxels for MRS. Localized 1H-MRS (PRESS TR/TE = 4,000/23 ms) were collected from HIPP and FC (volume 9.5 and 9.1 μl, respectively) as shown in **Figure 4A**, according to a quantitative protocol (Canese et al., 2012).

The following six metabolites were considered: *N*-acetyl-aspartate (NAA), myo-inositol (mINS), the sum of creatine and phosphocreatine (Cr + PCr), glutamate (Glu), glutamine (Gln), and total choline (tCho). Metabolite concentrations are expressed in mmol/L (mM). Moreover, we evaluated the NAA/Cr ratio and mINS/Cr.

### *In vivo* Microdialysis and HPLC Analysis

*In vivo* microdialysis was performed in awake, freely moving mice, as previously described (Cassano et al., 2012; Romano et al., 2014). Briefly, 6- and 12-month-old anesthetized mice (*n* = 5–6) were stereotaxically implanted with a CMA/7 guide cannula with a stylet (CMA Microdialysis, Stockholm, Sweden) into the vHIPP (anterior-posterior, −3.0 mm; lateral, +3.0 mm; ventral, −1.8 mm from bregma, **Figure 5A**), according to the stereotaxis atlas of Franklin and Paxinos (Franklin and Paxinos, 1997). Following a 2-day recovery period, the CMA/7 probe (6-kDa cutoff; 2 mm membrane length) was inserted and dialyses were carried out, in awake, freely moving mice, perfusing each probe with Krebs-Ringer phosphate (KRP) buffer at a flow rate of 1 μl/min. The constituents of the KRP buffer were (in mM) NaCl 145, KCl 2.7, MgCl_2_ 1, CaCl_2_ 2.4, and Na_2_HPO_4_ 2, buffered at pH 7.4. After a 2-h stabilization period, four baseline samples were collected every 20 min. Thereafter, the probes were perfused for 20 min with KCl (50 mM)-enriched KRP buffer, and then six samples were further collected with the previous KRP buffer. After completion of the microdialysis, the probe position was verified histologically, glutamate was quantified by HPLC coupled to fluorescence detection as previously described (Tomasini et al., 2002; Beggiato et al., 2020a,b).

### Statistical Analysis

The sample size was determined based on our previous experiments and using the free software G^*^Power version 3.1.9.2. All data are expressed as mean ± standard error of the mean (SEM). Within-group variability was analyzed through the Levene test for homogeneity of variances.

Data from mitochondrial bioenergetics and MRI/MRS scanning were analyzed using a 2-way analysis of variance (ANOVA) with genotype and treatment as the between variables. Dunnett's and Tukey's *post-hoc* tests were used where appropriate to perform multiple comparisons.

Regarding the microdialysis data, the overall basal glutamate levels were calculated as marginal means of the first five dialysate samples (from time −80 up to time 0) and were analyzed by two-way ANOVA with genotype and treatment as the between variables. The neurotransmitter release in response to depolarization (K^+^-stimulation) (from time 0 up to time 140) was analyzed using three-way ANOVA for repeated measures with genotype (3 × Tg-AD vs. non-Tg) and treatment (um-PEA vs. vehicle) as the between variable and time as the within the variable. Dunnett's and Tukey's *post-hoc* tests were used where appropriate to perform multiple comparisons.

The threshold for statistical significance was set at *p* < 0.05. The SPSS Statistics version 19 (IBM, Armonk, NY, USA) and GraphPad Prism 6 for Windows (GraphPad Software Inc., San Diego, CA, USA) were used to perform all the statistical analyses and represent graph data, respectively.

## Results

### Mitochondrial Respiration Activity Is Altered in Aged 3 × Tg-AD Mice: Effects of Chronic um-PEA Treatment

Oxygen consumption was detected in mitochondria freshly isolated from FC and HIPP of 6- and 12-month-old non-Tg and 3 × Tg-AD mice chronically treated with either vehicle or um-PEA. Statistical analyses are shown in Tables 1, 2.

**Table 1:** Statistical analyses of respiratory activity in mitochondria isolated from the frontal cortex.

		**Non-Tg + veh**	**Non-Tg + um-PEA**	**3 × Tg-AD + veh**	**3 × Tg-AD + um-PEA**	**F_**(1, 12)**_ G**	**F_**(1, 12)**_ T**	**F_**(1, 12)**_ G × T**
6-month-old	Complex I	State 4	3.02 ± 1.46	3.21 ± 1.55	3.03 ± 1.49	3.73 ± 2.43	0.089	0.250	0.082
		State 3	8.54 ± 4.42	9.04 ± 3.91	8.75 ± 4.39	9.62 ± 4.92	0.032	0.096	0.007
		RCI	2.96 ± 0.82	3.10 ± 0.74	3.04 ± 0.69	2.88 ± 0.80	0.033	0.001	0.154
	Complex II	State 4	8.81 ± 6.27	8.41 ± 4.10	8.06 ± 5.25	7.26 ± 4.32	0.141	0.056	0.006
		State 3	27.0 ± 17.1	24.9 ± 12.6	23.4 ± 14.7	22.3 ± 13.1	0.183	0.049	0.005
		RCI	3.16 ± 0.30	3.09 ± 0.91	2.98 ± 0.20	3.09 ± 0.19	0.130	0.006	0.130
12-month-old	Complex I	State 4	2.83 ± 1.08	2.91 ± 0.85	3.59 ± 2.80	3.28 ± 1.87	0.386	0.016	0.046
		State 3	7.21 ± 1.73	7.16 ± 1.94	**4.20** **±1.15↓**	**8.48** **±2.53↑**	0.789	4.943*	5.179*
		RCI	2.48 ± 0.50	2.61 ± 0.49	**1.80** **±0.23↓**	2.78 ± 0.51	4.125	11.36**	7.782*
	Complex II	State 4	9.01 ± 3.39	9.55 ± 2.18	9.82 ± 6.67	9.15 ± 3.57	0.009	0.001	0.080
		State 3	25.9 ± 13.5	26.1 ± 10.9	21.4 ± 6.85	25.7 ± 12.4	0.191	0.161	0.134
		RCI	2.73 ± 0.52	2.80 ± 0.74	2.62 ± 0.89	2.72 ± 0.35	0.083	0.067	0.002

Data are expressed as mean ± SEM of four experiments. Statistical differences were assessed by two-way ANOVA. Tukey's post-hoc test was used where appropriate to perform multiple comparisons. State 4 and state 3 are expressed as nmolO_2_/min/mg protein. ^*^p <0.05; ^**^p <0.01; ↑p <0.05 vs. 3xTg-AD + P; ↓p <0.05 vs. other study groups.

*RCI, respiratory control index; Veh, vehicle; umPEA, ultramicronized palmitoylethanolamide. Bold values are statistical significance*.

**Table 2:** Statistical analyses of respiratory activity in mitochondria isolated from the hippocampus.

		**Non-Tg + veh**	**Non-Tg + um-PEA**	**3 × Tg-AD + veh**	**3 × Tg-AD + um-PEA**	**F_**(1, 12)**_ G**	**F_**(1, 12)**_ T**	**F_**(1, 12)**_ G × T**
6-month-old	Complex I	State 4	4.94 ± 2.26	4.82 ± 1.79	5.50 ± 0.97	2.54 ± 1.08	1.136	3.642	3.096
		State 3	10.7 ± 3.22	9.94 ± 4.12	9.84 ± 3.62	6.17 ± 1.69	1.980	1.813	0.782
		RCI	2.33 ± 0.61	2.29 ± 0.77	1.78 ± 0.46	2.70 ± 1.03	0.035	1.384	1.648
	Complex II	State 4	9.45 ± 4.92	8.57 ± 5.18	9.16 ± 6.03	7.15 ± 4.09	0.112	0.321	0.049
		State 3	27.3 ± 20.0	25.9 ± 10.7	21.7 ± 12.3	21.5 ± 12.4	0.488	0.012	0.007
		RCI	2.66 ± 0.80	3.05 ± 0.49	2.47 ± 0.32	2.99 ± 0.05	0.254	3.363	0.069
12-month-old	Complex I	State 4	2.00 ± 0.79	2.31 ± 0.12	4.74 ± 1.40	5.62 ± 2.38	8.457*	1.590	0.817
		State 3	7.03 ± 2.35	7.44 ± 1.05	11.5 ± 4.41	12.3 ± 3.77	5.539*	0.390	0.154
		RCI	3.57 ± 0.28	2.98 ± 0.79	2.98 ± 0.84	2.39 ± 0.83	2.656	2.656	0.000
	Complex II	State 4	9.42 ± 6.60	10.9 ± 3.17	11.6 ± 4.07	12.4 ± 0.42	0.770	0.256	0.026
		State 3	23.7 ± 12.9	25.1 ± 8.95	23.9 ± 3.06	30.0 ± 6.18	0.353	0.764	0.300
		RCI	2.71 ± 0.50	2.68 ± 0.61	2.22 ± 0.72	2.42 ± 0.42	1.709	0.088	0.161

Data are expressed as mean ± SEM of four experiments. Statistical differences were assessed by two-way ANOVA. Tukey's post-hoc test was used where appropriate to perform multiple comparisons. State 4 and state 3 are expressed as nmolO_2_/min/mg protein. ^*^p <0.05.

*RCI, respiratory control index; Veh, vehicle; Um-PEA, ultramicronized palmitoylethanolamide. Bold values are statistical significance*.

**Figure 1:**
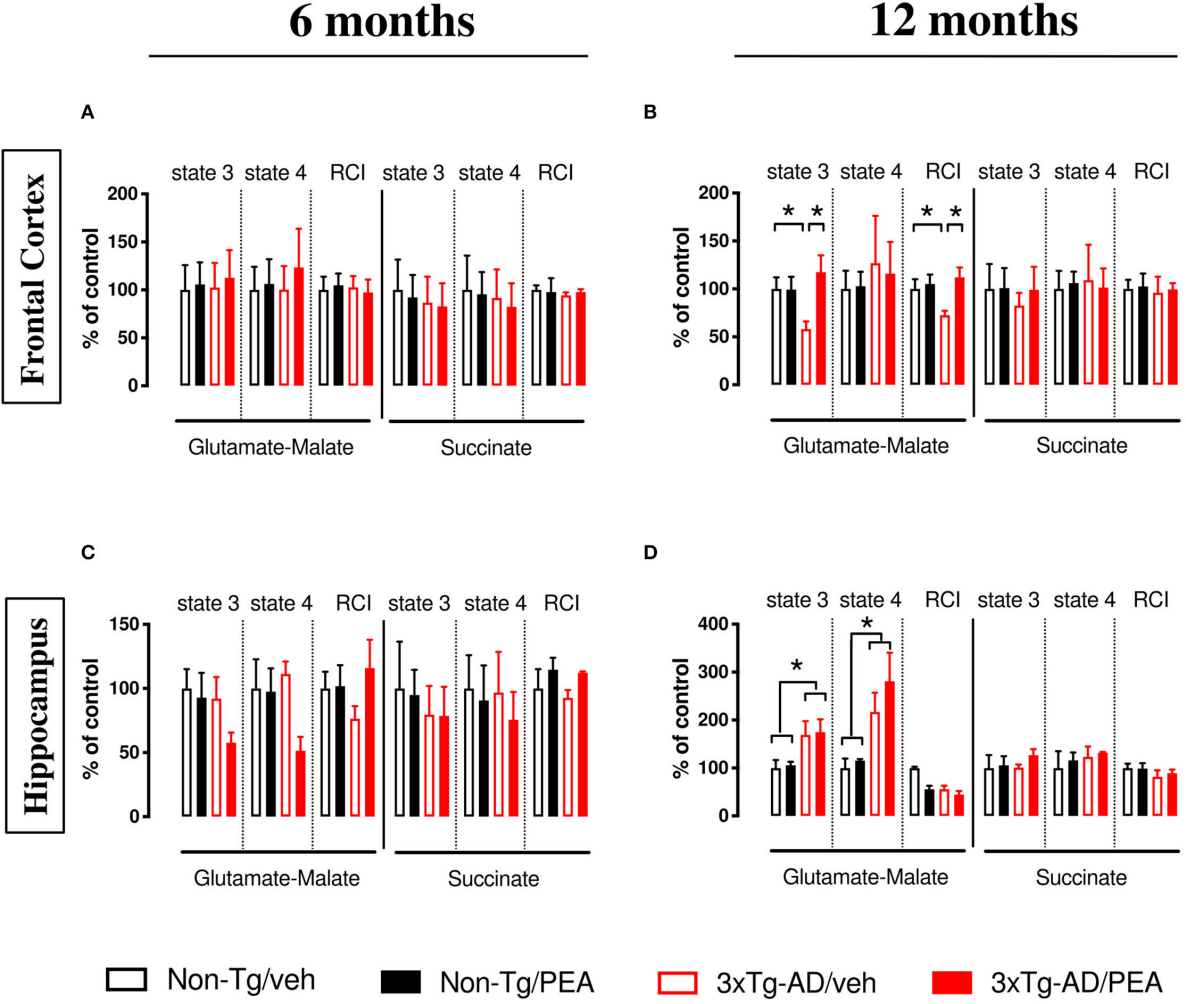
State 4 and state 3 mitochondrial respiratory activity and respiratory control index (RCI) measured in the frontal cortex **(A,B)**, and hippocampus **(C,D)** of non-Tg (black bars) and 3 × Tg-AD (red bars) 6-month-old **(A,C)** and 12-month-old **(B,D)** mice treated with vehicle (veh) (open bars) or um-PEA (filled bars), using complex I (glutamate-malate) and complex II (succinate) oxidative substrates. Data are expressed as a percentage of control ± SEM (*n* = 4). Statistical differences were assessed by two-way ANOVA and Tukey's *post-hoc* multiple comparisons test. *p < 0.05.

In 6-month-old mice, no significant difference was observed among experimental groups within the different brain regions (Figures 1A,C). Otherwise, when the NADH-generating substrates glutamate and malate were added to the mitochondrial preparations, a lower respiration activity in state 3 was detected in the FC of vehicle-treated 12-month-old 3 × Tg-AD compared to non-Tg mice (Figure 1B), whereas a significant increase in both state 4 and state 3 was detected in the HIPP compared to non-Tg mice (Figure 1D). The ratio between state 3 and state 4, named RCI, was decreased in the FC of vehicle-treated 3 × Tg-AD compared to non-Tg mice (Figure 1B), whereas it was unaffected in the HIPP of both genotypes, regardless of treatment (Figure 1D). um-PEA treatment significantly increased the state 3 respiration activity and the RCI in the FC of 3 × Tg-AD mice compared to the respective vehicle (Figure 1B).

The analysis of complex II respiration, using succinate as a substrate in the presence of rotenone, revealed that cortical (Figure 1B) and hippocampal (Figure 1D) mitochondria were unaffected by both genotype and treatment.

Finally, neither genotype nor treatment affected the respiration activity of the cerebellum when its mitochondria were incubated with either the complex I-linked substrates or complex II-linked substrates (data not shown).

### Chronic um-PEA Treatment Rescues the Impaired ATP Homeostasis in the Frontal Cortex of 3 × Tg-AD Mice

To investigate the effects of chronic um-PEA treatment on ATP homeostasis, F_o_F_1_-ATPase (complex V) activity, ADP/O ratio (which expresses the coupling between the phosphorylation activity and mitochondrial respiration), and ATP content were measured in both non-Tg and 3 × Tg-AD mice at two progressive stages of the disease (6 and 12 months of age).

**Figure 2:**
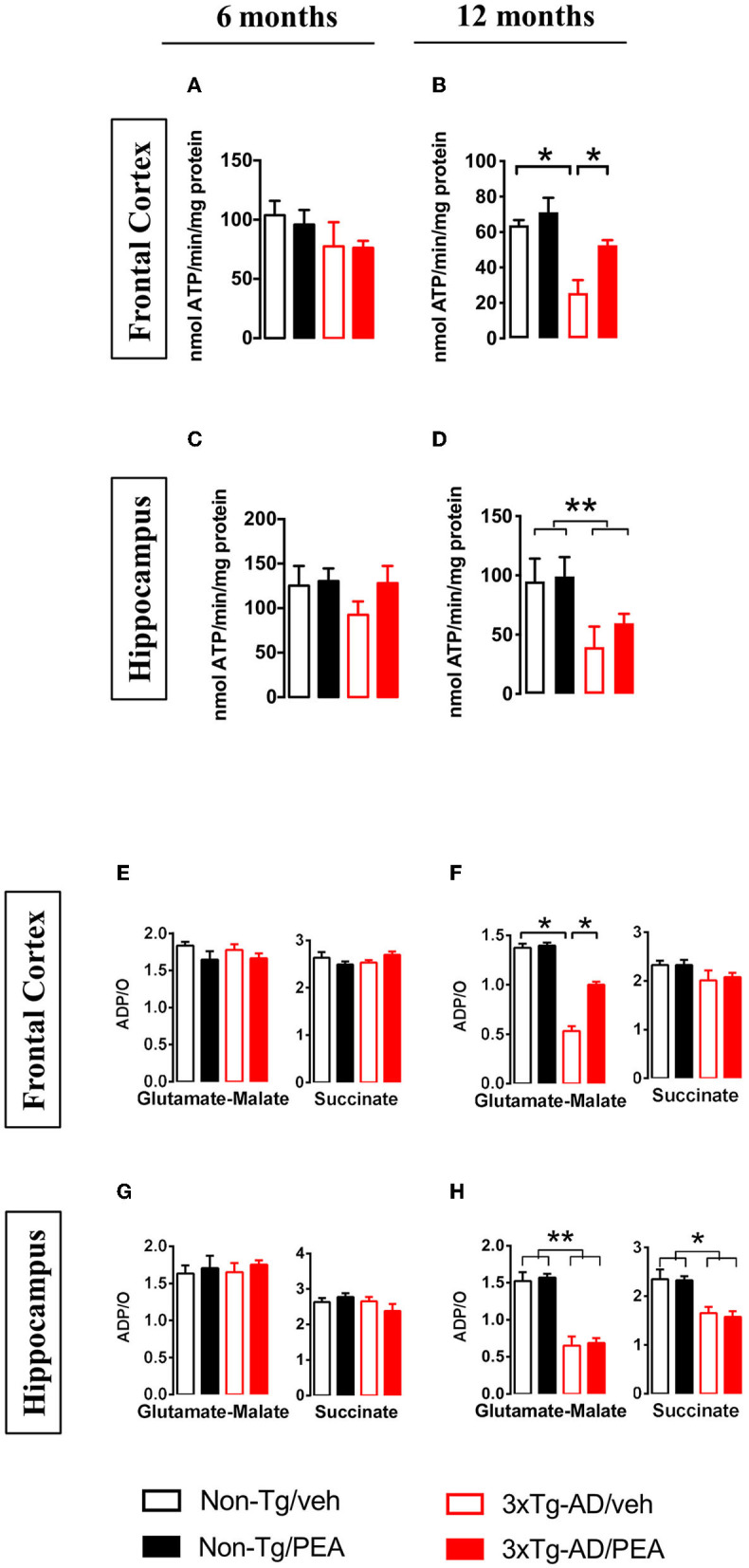
FoF_1_-ATPase activity **(A–D)** and ADP/O ratio **(E–H)** measured in mitochondria isolated from the frontal cortex **(A,B,E,F)**, and hippocampus **(C,D,G,H)** of non-Tg (black bars) and 3 × Tg-AD (red bars) 6-month-old **(A,C,E,G)** and 12-month-old **(B,D,F,H)** mice treated with vehicle (open bars) or um-PEA (filled bars). Data are expressed as mean ± SEM (*n* = 4). Statistical differences were assessed by two-way ANOVA and Tukey's *post-hoc* test. *p < 0.05; **p < 0.01.

Both the F_o_F_1_-ATPase activity and the ADP/O ratio were unaffected in mitochondria from FC (Figures 2A,E) and HIPP (Figures 2C,G) of both non-Tg and 3 × Tg-AD mice at 6 months of age. Different results were obtained for F_o_F_1_-ATPase activity and ADP/O ratio in the presence of glutamate-malate at 12 months of age, where a significant effect of genotype (*F*_(1, 12)_ = 15.59, *p* < 0.01; *F*_(1, 12)_ = 7.98, *p* < 0.05, respectively) and treatment (*F*_(1, 12)_ = 5.728, *p* < 0.05; *F*_(1, 12)_ = 4.956, *p* < 0.05, respectively) was observed in the FC (Figures 2B,F). Multiple *post-hoc* comparison showed a significantly lower F_o_F_1_-ATPase activity and ADP/O ratio for vehicle-treated 3 × Tg-AD mice compared to non-Tg mice that was counteracted by um-PEA treatment (Figures 2B,F). Regarding the HIPP, statistical analysis revealed only a significant main effect of genotype (*F*_(1, 12)_ = 6.001, *p* < 0.05; *F*_(1, 12)_ = 4.914, *p* < 0.05, respectively) (Figures 2D,H).

**Figure 3:**
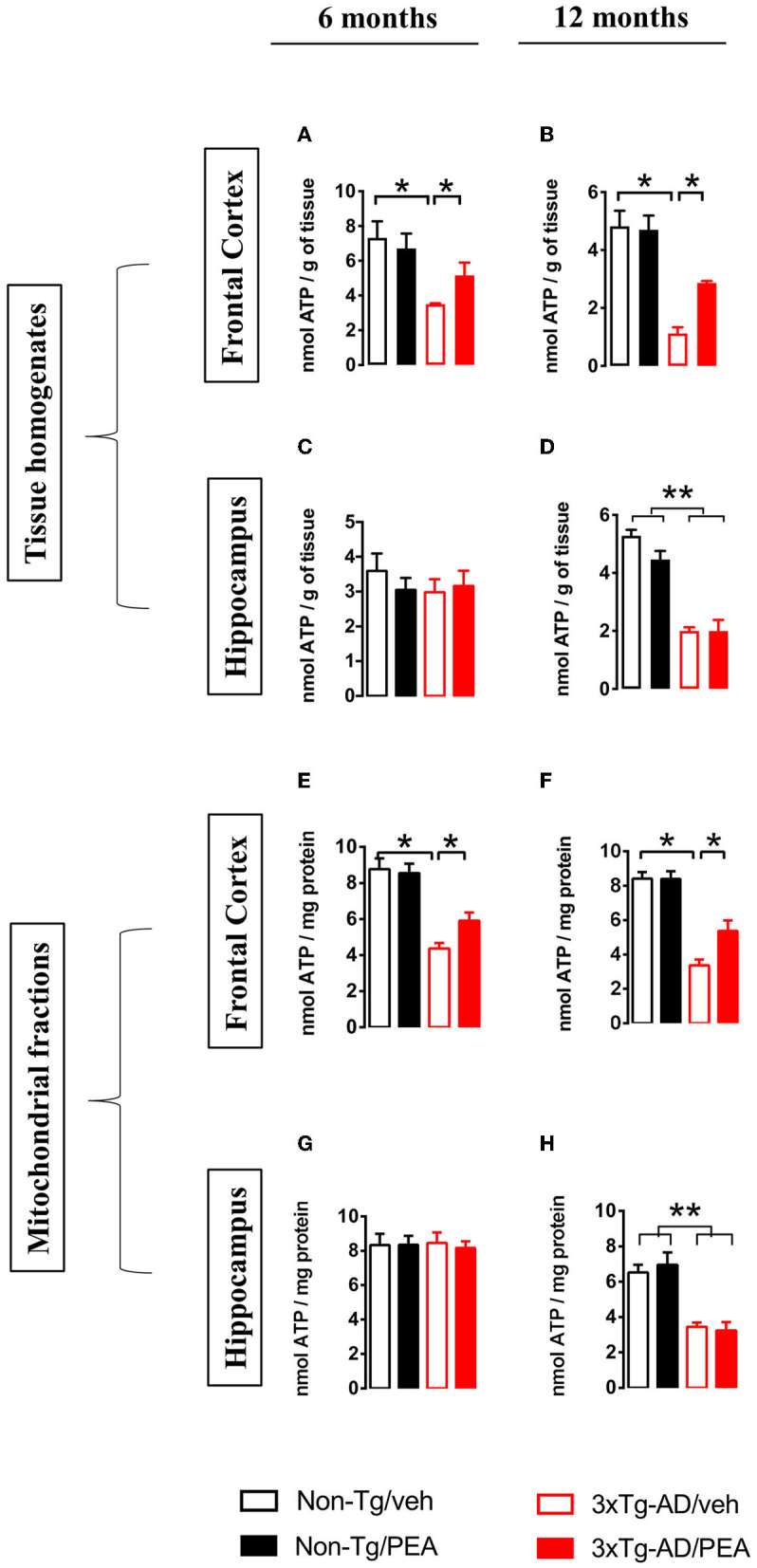
Total homogenate ATP content measured in tissue homogenates and mitochondria isolated from frontal cortex **(A,B,E,F)** and hippocampus **(C,D,G,H)** of non-Tg (black bars) and 3 × Tg-AD (red bars) 6-month-old **(A,C,E,G)** and 12-month-old **(B,D,F,H)** mice treated with vehicle (open bars) or um-PEA (filled bars). Data are expressed as mean ± SEM (*n* = 4). Statistical differences were assessed by two-way ANOVA and Tukey's *post-hoc* test. *p < 0.05; **p < 0.01.

To further evaluate the effects of chronic treatment on ATP homeostasis, we measured ATP levels in tissue homogenates and mitochondrial fractions from both brain regions. Statistical analysis showed a significant main effect of genotype [6 months: (*F*_(1, 12)_ = 9.738, *p* < 0.01); 12 months: (*F*_(1, 12)_ = 34.61, *p* < 0.001)] and treatment [6 months: (*F*_(1, 12)_ = 5.372, *p* < 0.05); 12 months: (*F*_(1, 12)_ = 6.152, *p* < 0.001] in the FC at both ages (Figures 3A,B,E,F). In particular, multiple *post-hoc* comparison showed a significant lower ATP content for vehicle-treated 3 × Tg-AD mice compared to non-Tg mice that was counteracted by um-PEA treatment (Figures 3A,B,E,F).

Regarding the ATP content in the HIPP, no difference was observed at 6 months of age, whereas a significant main effect of genotype (*F*_(1, 12)_ = 33.11, *p* < 0.001) was observed at 12 months of age (Figures 3C,D,G,H).

### MRI/MRS Study Shows Metabolic Changes in the Frontal Cortex and Hippocampus of 3 × Tg-AD Mice

**Figure 4:**
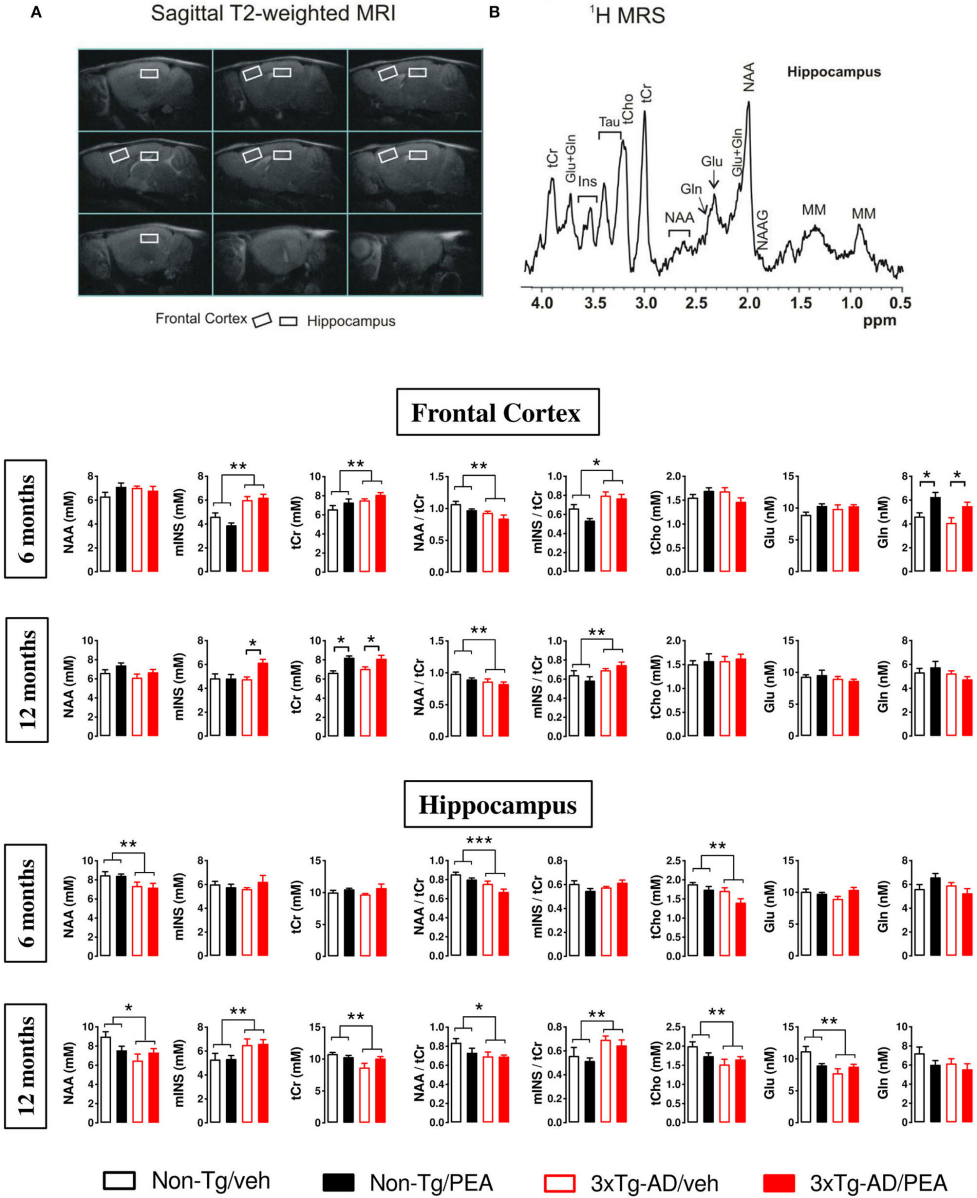
**(A)** MRI panel: example of *in vivo* fast spin-echo sagittal anatomical images [repetition time (TR)/echo time (TE) = 3,200/60 ms, consecutive slices]. Voxels localized in the frontal cortex and hippocampus are indicated by white rectangles. **(B)** MRS panel: examples of *in vivo* 1 H spectra (PRESS, TR/TE = 4,000/23 ms, NS = 256). Metabolite assignments: NAA, N-acetyl-aspartate; mINS, myo-inositol; tCr, total creatine; tCho, total choline; Glu, glutamate; Gln, glutamine. Histograms showing metabolites measured from the frontal cortex (upper panel) and hippocampus (lower panel) of non-Tg (black bars) and 3 × Tg-AD (red bars) 6-month-old and 12-month-old mice treated with vehicle (open bars) or um-PEA (filled bars). Data are expressed as mean ± SEM (*n* = 5–7). Statistical differences were assessed by two-way ANOVA and Tukey's *post-hoc* test. *p < 0.05; **p < 0.01.

Magnetic resonance imaging/spectroscopy was used to assess metabolic profiles of treated vs. untreated mice at 6 and 12 months of age. The quantitative results of all metabolite concentrations from FC and HIPP are reported in Figure 4. In the FC, two-way ANOVA analysis revealed no significant differences among groups in the NAA levels at both ages, whereas in the HIPP a main effect of genotype was observed at 6 (*F*_(1, 19)_ = 10.098, *p* < 0.01) and 12 months (F_(1, 22)_ = 5.820, *p* < 0.05).

Statistical analysis for mINS showed a significant main effect of genotype in the FC at 6 months of age (*F*_(1, 19)_ = 22.072, *p* < 0.01), whereas a significant main effect of treatment and genotype-by-treatment interaction effect was found at 12 months of age [(*F*_treatment(1, 25)_ = 5.040, *p* < 0.05); (*F*_genotype × *treatment*(1, 25)_ = 5.286, *p* < 0.05)]. *Post-hoc* comparisons showed a significantly higher mINS level in the um-PEA-treated compared to vehicle-treated 3 × Tg-AD mice. Hippocampal mINS levels showed no significant differences among groups at 6 months, whereas a significant main effect of genotype was observed at 12 months of age (*F*_(1, 23)_ = 6.502, *p* < 0.05).

Regarding the sum of creatine and phosphocreatine (Cr + PCr), named total creatine (tCr), two-way ANOVA analysis revealed in the FC a significant main effect of genotype at 6 months of age (*F*_(1, 20)_ = 5.616, *p* < 0.05), whereas a significant main effect of treatment was observed at 12 months (*F*_(1, 25)_ = 28.220, *p* < 0.01). *Post-hoc* comparisons within genotype showed that um-PEA treatment induced a significant increase in tCr levels compared to the vehicle-treated group. Moreover, hippocampal tCr levels showed no significant differences among groups at 6 months, whereas a significant main effect of genotype was observed at 12 months of age (*F*_(1, 20)_ = 5.843, *p* < 0.01).

Statistical analysis for NAA/tCr ratio revealed a significant main effect of genotype at both ages in the FC [6 months: (*F*_(1, 19)_ = 9.525, *p* < 0.01); 12 months: (*F*_(1, 25)_ = 9.111, *p* < 0.01)], as well as in the HIPP [6 months: (*F*_(1, 19)_ = 22.910, *p* < 0.01); 12 months: (*F*_(1, 18)_ = 5.101, *p* < 0.05)]. Regarding the mINS/tCr ratio, two-way ANOVA analysis showed a significant main effect of genotype at both ages in the FC [6 months: (*F*_(1, 17)_ = 18.179, *p* < 0.05); 12 months: (*F*_(1, 21)_ = 10.820, *p* < 0.01)], as well as at 12 months of age in the HIPP (*F*_(1, 18)_ = 9.217, *p* < 0.01), whereas no significant differences among groups were found at 6 months in the HIPP.

Statistical analysis for tCho showed no significant differences among groups in the cortical levels at both ages, whereas in the HIPP a main effect of genotype was observed at 6 (*F*_(1, 19)_ = 14.329, *p* < 0.01) and 12 months of age (*F*_(1, 24)_ = 6.007, *p* < 0.05).

Glu concentrations showed no differences among groups at both ages in the FC, as well as at 6 months in the HIPP; a significant main effect of genotype was observed at 12 months of age in the HIPP (*F*_(1, 22)_ = 5.619, *p* < 0.05). Statistical analysis for Gln revealed a significant main effect of treatment at 6 months of age in the FC (*F*_(1, 18)_ = 14.586, *p* < 0.01). *Post-hoc* comparisons within genotype showed that um-PEA treatment induced a significant increase in Gln levels compared to the vehicle-treated group. Moreover, no significant differences among groups were observed at 12 months in the FC, as well as in the HIPP at both ages.

### Chronic um-PEA Treatment Does Not Ameliorate the Impaired K^+^-Evoked Release of Glutamate in the Ventral Hippocampus of 3 × Tg-AD Mice

**Figure 5:**
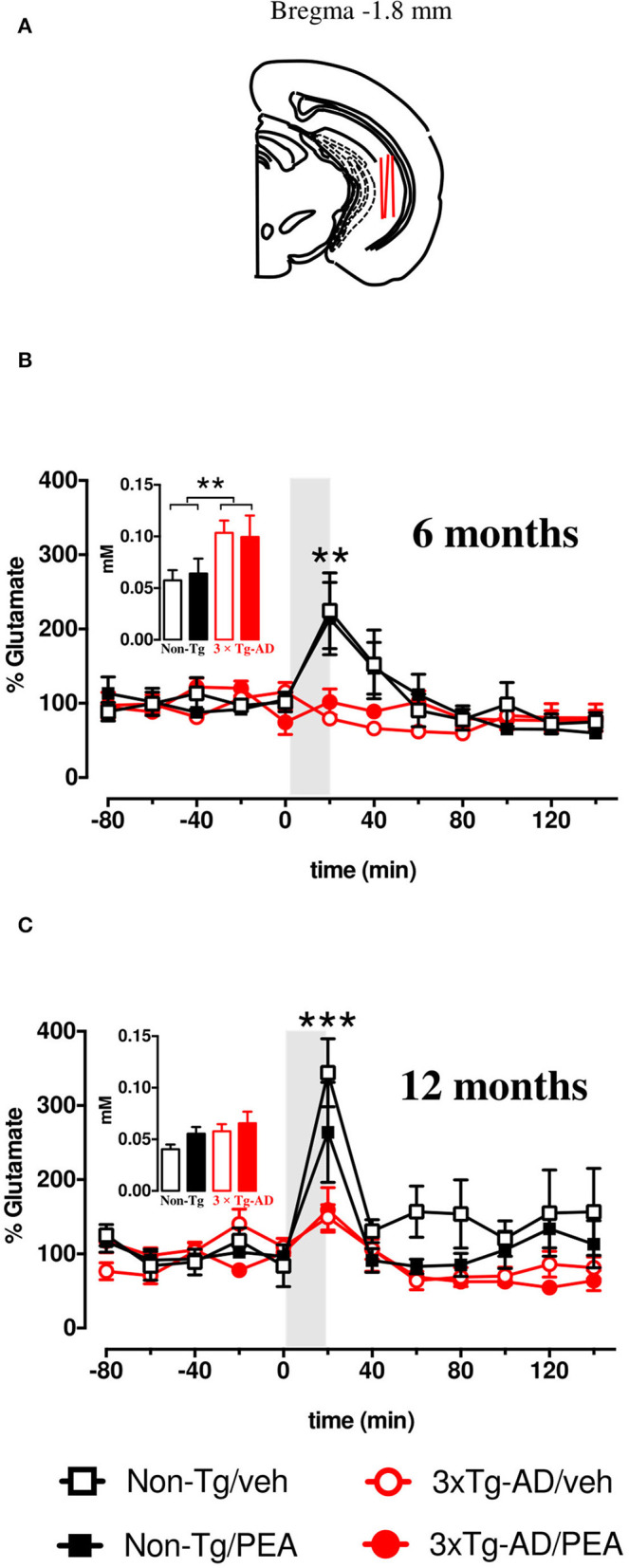
**(A)** Brain diagrams illustrating the average sites (red lines) where microdialysis probes were implanted and where the representative microphotographs were taken. K^+^-evoked release of glutamate in the ventral hippocampus of 6- **(B)** and 12-month-old **(C)** freely moving non-Tg (squares) and 3 × Tg-AD (circles) mice chronically treated with vehicle (empty squares and empty circles, respectively) or um-PEA (black squares and red circles, respectively); gray areas indicate the perfusion with KCl-enriched (50 mM) Krebs-Ringer phosphate (KRP) buffer. Histograms represent average baseline levels (marginal means of five consecutive dialysates: from time −80 up to time 0) observed in non-Tg (black bars) and 3 × Tg-AD (red bars) mice treated with vehicle (open bars) and um-PEA (filled bars). Data are expressed as a percentage of control ± SEM (*n* = 5–6). Statistical differences were assessed by three-way ANOVA and Dunnett's multiple comparison test. **p < 0.01; ***p < 0.001 vs. last baseline within the same group.

As we have previously published (Scuderi et al., 2018), the basal extracellular glutamate levels in the vHIPP of 6-month-old 3 × Tg-AD mice were significantly higher compared to age-matched non-Tg mice, whereas no significant genotype-related difference in basal glutamate levels was observed in 12-month-old mice. Moreover, chronic um-PEA treatment did not affect the basal output of glutamate at both ages (Scuderi et al., 2018). The overall basal glutamate levels are recapitulated in the insets of Figure 5.

After five consecutive samples, the basal extracellular levels of glutamate reached a steady state in the vHIPP of all mice. To address the impact of chronic um-PEA treatment on the impulse-driven glutamate release in the vHIPP of non-Tg and 3 × Tg-AD mice, at both mild (6 months of age) and severe (12 months of age) stages of pathology, the probes were perfused with K+-enriched KRP buffer (containing KCl 50 mM) for 20 min (stimulated condition). Under the stimulated condition, the glutamate extracellular levels of non-Tg mice were increased due to the ability of neurons to increase their synaptic activity in response to depolarization. In this study, we observed the total lack of response to K+-stimulation in the vHIPP of 3 × Tg-AD mice compared to the non-Tg group, at both 6 and 12 months of age. Moreover, chronic treatment of PEA did not ameliorate the impaired K+-evoked output of glutamate neurotransmission in the 6- and 12-month-old 3 × Tg-AD mice (Figures 5A,B). The results of the statistical analyses performed by three-way ANOVA are reported in Table 3.

**Table 3:** Statistical analyses of K^+^-evoked release of glutamate in the ventral hippocampus of 3 × Tg-AD mice and non-Tg mice.

	**6 months of age**			**12 months of age**		
	**Df**	**F**	**P**	**df**	**F**	**P**
Genotype (G)	1, 21	5,845	**0,0248**	1, 18	13,244	**0,0019**
Treatment (Tr)	1, 21	0,004	0,9517	1, 18	2,666	0.1199
G × Tr	1, 21	0,252	0,6209	1, 18	1,353	0,2599
Time (T)	7, 147	9,910	**<0,0001**	7, 126	19,526	**<0,0001**
T × G	7, 147	7,446	**<0,0001**	7, 126	5,446	**<0,0001**
T × Tr	7, 147	0,924	0,495	7, 126	0.433	0,8802
T × G × Tr	7, 147	0,477	0,8500	7, 126	0.943	0,4761

*Statistical differences were assessed by three-way ANOVA for repeated measures with genotype (G) (3 × Tg-AD vs. non-Tg) and treatment (Tr) (um-PEA vs. vehicle) as the between variable and time (T) as the within variable. Dunnett's and Tukey's post-hoc tests were used where appropriate to perform multiple comparisons. Bold values are statistical significance*.

## Discussion

The most novel findings of our study are the demonstration that chronic um-PEA treatment can ameliorate the complex-I respiration rate, the F_o_F_1_-ATPase (complex V) activity, as well as ATP content in the cortical mitochondria from 3 × Tg-AD mice. Otherwise, mitochondrial bioenergetics, as well as the release of glutamate after depolarization were not ameliorated by um-PEA treatment in the HIPP of both young and adult 3 × Tg-AD mice. More interestingly, progressive age- and pathology-related changes were observed in the cortical and hippocampal metabolism that closely mimic the alterations observed in the human AD brain; these metabolic alterations were not affected by chronic um-PEA treatment.

We revealed for the first time that (i) AD-like pathology differently affects mitochondrial bioenergetics in the FC and HIPP and (ii) um-PEA treatment does not promote significant ameliorations simultaneously in those brain regions of 3 × Tg-AD mice. This is in line with the temporal- and regional-specific development of AD neuropathology in the brain of 3 × Tg-AD mice, which closely mimics their development in the human AD brain (Oddo et al., 2003a,b; Hirata-Fukae et al., 2008).

Compared with many studies, we adopted a longitudinal study design with a sufficiently long observation period that allowed us to assess early alterations and changes over time in both non-transgenic and 3 × Tg-AD mice. This study further extends the knowledge of the molecular modification during both normal aging and the progression of AD-like pathology in our murine model of AD (Oddo et al., 2003a,b). Moreover, we analyzed the modulatory effect of PEA on pathways and factors consistently associated with AD-like pathology and symptoms. In fact, we selected two different subsets of 3 × Tg-AD mice, which allowed us to investigate the therapeutic potential of um-PEA in restraining the development of AD-like pathology at mild and severe stages, focusing on the mitochondrial bioenergetics, cerebral metabolism, and glutamatergic transmission. Regarding mitochondria and glutamate, we have previously demonstrated that deficits of glutamatergic transmission and mitochondrial dysfunction coexist in the FC and HIPP of 18-month-old 3 × Tg-AD mice, which show a substantial number of amyloid plaques and tau pathology; the HIPP was the most affected area at that age (Cassano et al., 2012).

Despite accumulating evidence supporting the link between inflammation, mitochondria, and metabolism, several important questions remain unanswered. Furthermore, there are no studies addressing the effects of PEA on mitochondria bioenergetics in *in vivo* experimental models of AD, and only one study, to our knowledge, has assessed *in vivo* the effects of PEA on mitochondrial dysfunction observed in central nervous system pathological conditions (Cristiano et al., 2018). In this regard, Cristiano and colleagues found that PEA treatment improves hippocampal mitochondrial function and reduces oxidative stress in a genetic murine model (*BTBR T* + *tf/J*), which exhibits a behavioral phenotype of autism spectrum disorder. In particular, 10-day treatment with um-PEA was able to (i) restore hippocampal mitochondrial state 3 respiration using succinate as substrate, (ii) increase superoxide dismutase (SOD) activity, (iii) counteract ROS, and (iv) decline the energy efficiency, as evidenced by the decreased degree of coupling (Cristiano et al., 2018). Together, these findings demonstrate that PEA counteracts mitochondrial dysfunction and helps rescue brain energy metabolism during pathological states, balancing ROS production/antioxidant defenses, and limiting oxidative stress. Moreover, PEA-treated BTRB mice showed a low activation of pro-inflammatory cytokines at hippocampal and serum levels (Cristiano et al., 2018). Likewise, we have previously observed that 3-month treatment of um-PEA almost completely abolishes the increase in inflammatory markers observed in 6-month-old 3 × Tg-AD mice and suppresses the expression of proinflammatory mediators, including interleukin (IL)-1β, IL-16, IL-5, monocyte chemotactic protein 5 (MCP-5), and macrophage colony-stimulating factor (M-CSF), but not inducible nitric oxide synthase (iNOS) and tumor necrosis factor (TNF), while enhancing the anti-inflammatory IL-10 (Scuderi et al., 2018).

In this study, 6-month-old 3 × Tg-AD mice with a mild AD-like pathology showed a significant reduction of ATP content in the FC that was counteracted by um-PEA treatment. Otherwise, at 12 months of age, together with severe hallmarks of AD-like pathology, mitochondrial alterations were more evident and regional-specific. In fact, cortical mitochondria of 3 × Tg-AD mice exhibited a reduced respiratory capacity, which might partially block electron flow within the respiratory chain and consequently increase oxidative stress; this effect was counteracted by um-PEA. The reduced respiratory activity was not observed after incubation with a complex II-linked substrate. The same pattern of alterations was observed in the cortical mitochondria of 18-month-old 3 × Tg-AD mice, which present diffuse extracellular Aβ deposits and extensive human tau immunoreactivity (Cassano et al., 2012). Different from the latter age, at 12 months the reduced oxygen consumption observed in the transgenic cortical preparations was accompanied by alterations of F_o_F_1_-ATPase activity and ATP content that were counteracted by um-PEA treatment.

Regarding the HIPP, 12-month-old 3 × Tg-AD mice showed the same mitochondrial dysfunctions previously observed with mice at 18 months of age (Cassano et al., 2012). In fact, here we confirmed a significant increase in state 3 and state 4 respiration rates with complex I-linked substrates and a significant impairment of ATP homeostasis. Different from FC, the pharmacological treatment with um-PEA did not ameliorate the hippocampal mitochondrial bioenergetics. Taken together, these findings suggest that um-PEA counteracts mitochondrial dysfunction, helps rescue brain energy metabolism during pathological states only in the FC, and such discrepancy might be due to the difference in Aβ/tau-linked alterations observed between these brain regions. In fact, it has been documented that Aβ and tau pathology differently impact brain regions, with HIPP showing more severe alterations compared to FC (Oddo et al., 2003a,b; Cassano et al., 2012; Bellanti et al., 2017). However, other mechanisms could underlie the regional-specific effect of um-PEA on mitochondrial bioenergetics and other experiments will be necessary to explain this phenomenon.

Brain structures undergo drastic modifications during aging, becoming progressively less interconnected and undergoing several metabolic and structural changes. A clinical scanner by MRS may serve to identify patients with AD before clinical symptom onset to help distinguish AD from other neurodegenerative disorders, as well as evaluate treatment effects.

In this regard, clinical studies demonstrated that NAA, a marker of neuronal integrity, can be detected by MRS and used to differentiate normal aging and pathological dementia (Ackl et al., 2005; Jessen et al., 2005; den Heijer et al., 2006). Besides decreased levels of NAA, several clinical studies demonstrated that mINS, a marker of glial activation, was increased in patients with AD (Glanville et al., 1989; Miller et al., 1993; Bitsch et al., 1999; Jessen et al., 2000; Kantarci et al., 2000; Huang et al., 2001).

Herein, we performed for the first time a comprehensive evaluation of six brain metabolites in the areas most involved in AD-like pathology. Interestingly, HIPP of 3 × Tg-AD mice was characterized by lower concentrations of NAA and increased mINS, as seen in human patients with AD (Glanville et al., 1989; Klunk et al., 1992; Miller et al., 1993; Bitsch et al., 1999; Jessen et al., 2000; Kantarci et al., 2000, 2007; Huang et al., 2001; Cheng et al., 2002; Schott et al., 2010). In particular, NAA reduction was observed at both ages (6 and 12 months), whereas mINS increased gradually with mouse age. Similar results were observed with APP/PS1 double transgenic mice, which is characterized by the early appearance of Aβ plaques, neuronal degeneration, and synaptic loss in the brain (Marjanska et al., 2005; Chen et al., 2009). The authors found that NAA was significantly reduced both in the FC and HIPP of 5-month-old APP/PS1 transgenic mice when pathology showed the formation of sparse Aβ plaques in these areas, and the number of neurons decreased. As the age of the mice increased, the NAA decrease was continuously accompanied by the increase in both mINS and the number of Aβ plaques (Marjanska et al., 2005; Chen et al., 2009). In our 3 × Tg-AD mice, we found an increase in mINS by age only in the HIPP, but not in the FC, where a significantly higher concentration was observed only at 6 months of age compared to the non-Tg group.

Other results were obtained in the FC with different transgenic AD mice. In fact, no increase in mINS in the FC was observed in the transgenic mouse line PS2APP [PS2N141I × APP(swe)], which develops an age-related cognitive decline associated with severe amyloidosis (Von et al., 2005). Therefore, further studies need to be performed to justify the discrepancy existing in the literature, which may be attributed to the differences in the experimental parameters and animal models of AD-like pathology.

Our longitudinal study revealed a significant decrease in tCr only in the HIPP of 12-month-old 3 × Tg-AD mice. This metabolic profile, together with lower NAA, is a clear indicator of the age-related hypometabolism in the HIPP of 3 × Tg-AD mice, further suggesting that HIPP is the most affected area at that age (Cassano et al., 2012; Scuderi et al., 2018).

A decrease in NAA/Cr ratio accompanied by an increased mINS/Cr ratio occurs in patients with AD; the latter temporal progression of metabolite abnormalities in patients with AD correlates with the progression of the Aβ/tau-linked alterations (Glanville et al., 1989; Klunk et al., 1992; Miller et al., 1993; Bitsch et al., 1999; Jessen et al., 2000; Kantarci et al., 2000, 2007; Huang et al., 2001; Cheng et al., 2002; Schott et al., 2010). Interestingly, we found a similar pattern of changes in the metabolite ratio both in the FC and HIPP, as seen in human patients with AD.

The role of Cho metabolite in AD is more controversial since several clinical studies demonstrated an increase (Meyerhoff et al., 1994; Pfefferbaum et al., 1999; Kantarci et al., 2007), others no change (Moats et al., 1994; Schuff et al., 1997; Rose et al., 1999; Krishnan et al., 2003), whereas a decrease was reported in one study (Chantal et al., 2002). In our study, we observed a significant decrease of tCho only in the HIPP of both 6- and 12-month-old mice. The Cho cytosolic concentration derives from the breakdown products of phosphatidylcholine (Schuff et al., 1997). Therefore, it has been proposed that catabolism of the phospholipid membrane bilayer allows AD subjects to produce choline to compensate for declining acetylcholine (Wurtman et al., 1985).

Although Glu is the principal excitatory neurotransmitter involved in learning and memory, it has been less well investigated by MRS/MRI. In this regard, few clinical studies revealed a reduction of Glu levels in different brain regions of patients with AD (Antuono et al., 2001; Hattori et al., 2002; Rupsingh et al., 2011) that correlates with the severe reduction of the cerebral metabolic rate for glucose (Mosconi et al., 2008; Bedse et al., 2015; Barone et al., 2016). In fact, glutamate neurotransmission is carried out by a glial-neuronal process that includes the oxidation of glucose and the ATP-dependent glutamine-glutamate cycle. A total of 80–90% of total cerebral glucose usage is attributable to the energy requirements of glutamatergic neurotransmission (Magistretti et al., 1999; Gatta et al., 2016; Pardeshi et al., 2017; Tramutola et al., 2018; Sharma et al., 2019; Bukke et al., 2020a,b). The neurochemical changes obtained from *in vivo* human MRS studies agree with the results observed in preclinical studies. In fact, the analysis of cortex and HIPP in APP-PS1 mice showed an age-dependent reduction of Glu levels, which correlates with increasing brain amyloidosis (Marjanska et al., 2005). Similar findings were observed in the cortex of APP (Dedeoglu et al., 2004) and PS2APP mice (Von et al., 2005). This is in line with the Glu spectroscopy findings between human AD subjects and the three transgenic mouse models of AD.

In our study, we demonstrated a temporal- and regional-specific alteration of Glu in 3 × Tg-AD mice with the HIPP the only affected area. In particular, at 12 months of age, we found a significant reduction (−38%) of Glu in the HIPP of vehicle-treated 3 × Tg-AD vs. vehicle-treated non-Tg mice. These results, observed in the vehicle-treated 3 × Tg-AD mice, together with lower hippocampal NAA (−45%), tCr (−24%), and tCho (−31%), further support the evidence of an age-related hypometabolism in the HIPP. MRS analysis might be of interest also to depict how neural correlates do change along with pharmacological treatment. In this regard, in our experimental condition, we did not observe any gross treatment-dependent differences between genotypes.

To investigate whether the decrease in hippocampal glutamate levels in the metabolic pool was paralleled by alterations in the glutamatergic nerve terminals, the pattern of glutamate release in basal condition, and in response to K^+^-stimulation was explored by microdialysis sampling in the vHIPP of 6- and 12-month-old mice. We have previously demonstrated that the basal release of glutamate was significantly increased (+80%) at 6 months of age whereas a trend toward an increase (+43%) was observed in 12-month-old 3 × Tg-AD compared to non-Tg mice (Scuderi et al., 2018). Here, we demonstrated that at both ages the depolarization-evoked glutamate release resulted completely disrupted in 3 × Tg-AD compared to non-Tg mice, thus suggesting that the deficit of glutamate metabolic pool observed by MRS might sustain, at least in part (at 12 months of age), the decrease of extracellular glutamate concentration in mutant mice.

## Conclusion

Our results not only elucidate the temporal correlation among mitochondrion, metabolic alterations, and glutamatergic dysfunction but also, in future studies, allow the assessment of putative therapeutic strategies. However, this study presents some limitations. For instance, additional measurements would allow performing a tight correlation between molecular markers of bioenergetics dysfunction, for instance, hydrogen peroxide production, proton leak or membrane potential, and metabolic alterations. Finally, this study sheds additional light on the effects of PEA treatment, displaying selective bioenergetic effects. At present, the use of a PEA treatment may represent a possible novel frontier for the future clinical treatment of AD.

## Data Availability Statement

The raw data supporting the conclusions of this article will be made available by the authors, without undue reservation.

## Ethics Statement

The animal study was reviewed and approved by Ethics Committee of the University of Foggia.

## Author Contributions

TC, CS, LS, and GS designed the research. FB, VB, MA, RV, GP, RC, and SB performed the research. FB, RC, GV, GS, and TC analyzed data and interpreted the results. FB and TC wrote the manuscript. All authors contributed to the article and approved the submitted version.

## Funding

This project was supported by the Italian Ministry for Education, University and Research (PRIN to LS and GV), and the Department of Clinical and Experimental Medicine (Dipartimenti di eccellenza – Legge 232/2016). This paper/manuscript/study has been published with the financial support of the Department of Medical and Surgical Sciences of the University of Foggia.

## Conflict of Interest

The authors declare that the research was conducted in the absence of any commercial or financial relationships that could be construed as a potential conflict of interest.

## Publisher's Note

All claims expressed in this article are solely those of the authors and do not necessarily represent those of their affiliated organizations, or those of the publisher, the editors and the reviewers. Any product that may be evaluated in this article, or claim that may be made by its manufacturer, is not guaranteed or endorsed by the publisher.
